# Monitoring antihypertensive drug concentrations to determine nonadherence in hypertensive patients with or without a kidney transplant

**DOI:** 10.1097/HJH.0000000000003459

**Published:** 2023-05-06

**Authors:** Laura E.J. Peeters, Dennis A. Hesselink, Melvin Lafeber, David Severs, Martijn W.F. van den Hoogen, Michelle A.H. Sonneveld, Christian R.B. Ramakers, Soma Bahmany, Teun van Gelder, Birgit C.P. Koch, Jorie Versmissen

**Affiliations:** a Erasmus MC, University Medical Center Rotterdam, Department of Hospital Pharmacy; b Erasmus MC, University Medical Center Rotterdam, Department of Internal Medicine, Division of Vascular Medicine; c Erasmus MC, University Medical Center Rotterdam, Department of Internal Medicine, Division of Nephrology and Transplantation; d Erasmus MC, University Medical Center Rotterdam, Erasmus MC Transplant Institute; e Erasmus MC, University Medical Center Rotterdam, Department of Clinical Chemistry, Rotterdam, the Netherlands

**Keywords:** adherence, hypertension, kidney transplantation, plasma, prevalence, therapeutic drug monitoring

## Abstract

**Background:**

Nonadherence to antihypertensive drugs (AHDs) is a major contributor to pseudo-resistant hypertension. The primary objective of this study was to determine the prevalence of nonadherence to AHDs among patients visiting the nephrology and vascular outpatient clinics.

**Methods:**

Patients were eligible to participate in this prospective observational study if they used at least two AHDs that could be measured with a validated UHPLC-MS/MS method and had an office blood pressure at least 140 and/or at least 90 mmHg. For resistant hypertension, included patients had to use at least three AHDs including a diuretic or four AHDs. Adherence was assessed by measuring drug concentrations in blood. The complete absence of drug in blood was defined as nonadherence. A posthoc analysis was performed to determine the influence of a having a kidney transplant on the adherence rates.

**Results:**

One hundred and forty-two patients were included of whom 66 patients fulfilled the definition of resistant hypertension. The overall adherence rate to AHDs was 78.2% (*n* = 111 patients), with the highest adherence rate for irbesartan (100%, *n* = 9) and lowest adherence rate for bumetanide (*n* = 69%, *n* = 13). In further analysis, only kidney transplantation could be identified as an important factor for adherence (adjusted odds ratio = 3.35; 95% confidence interval 1.23–9.09). A posthoc analysis showed that patients with a kidney transplant were more likely to be adherent to AHDs (non-KT cohort 64.0% vs. KT-cohort 85.7%, χ^2^(2) = 10.34, *P* = 0.006).

**Conclusion:**

The adherence rate to AHDs in hypertensive patients was high (78.2%) and even higher after a kidney transplant (85.7%). Furthermore, patients after kidney transplant had a lower risk of being nonadherent to AHDs.

## INTRODUCTION

Identification of nonadherence to antihypertensive drugs (AHDs) in patients with hypertension is important to understand in which patients strategies to improve adherence need to be applied, while preventing referral for experimental invasive procedures [1–3]. Nonadherence is thought to be a major contributor to so-called (therapy-)resistant hypertension, which is associated with a higher risk of cardiovascular events, stroke and kidney injury [2,4].

Patients are categorized as having resistant hypertension when they have office values more than 140 mmHg SBP and more than 90 mmHg DBP despite the prescription of three AHDs including a diuretic or four or more AHDs, all in the most optimal dose [2]. Resistant hypertension due to nonadherence is often referred to as ‘pseudo-resistance’, as blood pressure (BP) control can be improved with better adherence [4,5].

Unfortunately, identifying nonadherence is challenging due to two important issues. The first one is so-called ‘white-coat’ adherence [6]. White-coat adherence makes patients improve their adherence to drugs when they are aware of measurements to assess drug intake. This would give an overestimation of the adherence rate and makes the actual prevalence of nonadherence to drugs hard to establish [3]. Therefore, white-coat adherence is of major influence when investigating adherence in a research setting wherein informed consent is required before performing any research-related medical act.

A second issue that contributes to the identification challenge is the availability of an accurate method to assess nonadherence to AHDs [7]. Several methods are available, but all have their pro's and con's [6]. One of the most reliable methods to identify nonadherence is measurement of drug concentrations [8]. Drug concentrations are often determined in blood and have to be collected by means of a venipuncture, which is an extra burden for the patient [9]. An easy, patient-friendly method is to use residual blood to decrease the burden and possibly reduce white-coat adherence.

Because of these mentioned challenges, the actual adherence rates in patients with resistant hypertension are still largely unknown and differ between studies depending on the used methods and definitions [7]. Therefore, the primary objective of this study was to determine the adherence to AHDs in patients visiting the nephrology and vascular outpatient clinics by measuring AHD concentrations in blood from residual material.

## MATERIALS AND METHODS

RHYME-AD (Resistant Hypertension: MEasure Antihypertensive Drugs) was an uncontrolled prospective observational study to determine nonadherence to AHDs by measuring drug concentrations in a hypertensive population.

### Participants

#### Inclusion and exclusion criteria

Patients were recruited from the vascular outpatient clinic for hypertension and the nephrology outpatient clinic including patients with a kidney transplantation of the Erasmus MC, University Medical Center Rotterdam, Rotterdam, The Netherlands, between June 2020 and July 2021. The Erasmus MC is a tertiary university hospital and one out of seven hospitals in the Netherlands that performs kidney transplants. Patients were eligible for participation if they were 18 years or older, had an office BP at least 140 and/or at least 90 mmHg and were treated with at least two AHDs for which an ultra-high-performance liquid chromatography-tandem mass spectrometry (UPLC-MS/MS) method was developed and validated to accurately measure drug concentrations up until 24 h after intake. This method included the following AHDs and four of their active [metabolites]: enalapril [enalaprilate], perindopril [perindoprilate], irbesartan, valsartan, losartan [losartan-carboxylic acid], bumetanide, spironolactone [canrenone], amlodipine, barnidipine, nifedipine, metoprolol and doxazosin [10,11].

Patients were excluded from participation if they were not able or willing to give informed consent or had an estimated eGFR less than 15 ml/min per 1.73 m^2^. Patients with renal failure were excluded because AHDs work differently in patients with end-stage renal failure, and therefore, therapy will be different [12]. This makes them a totally different population compared with patients with an eGFR more than 15 ml/min per 1.73 m^2^. Furthermore, patients were not included if they visited the outpatient clinic for the first time or if secondary causes of hypertension were expected but not confirmed yet.

This study was approved by the local medical ethical committee of the Erasmus Medical Center, Rotterdam, the Netherlands (MEC-2020-0096). All patients provided written informed consent.

### Study design

Eligible patients were asked by their treating physician to participate in this study and were provided with a patient information leaflet (PIL) if the patient was interested in participation and informed consent could be signed directly if the physician provided the patients with all the information. Each patient who received a PIL was reported by the physician to the coordinating researcher. If written consent was not received within 2 weeks after issuance of the PIL, patients were called by the coordinating researcher to give additional information. After written informed consent was provided, physicians were instructed to add a short-term bio bank request to each clinically ordered blood sample. With this added request, an aliquot of lithium heparin (BD Vacutainer Barricor; Becton Dickinson (BD). Heidelberg, Germany) was stored for 6 months at −20°C enabling the researchers to retrieve the residual material for AHD testing. With this approach, the patients were not informed specifically when the measurement would take place. Participants were labelled as having (apparent) resistant hypertension when they had a prescription of three AHDs including a diuretic or at least four AHDs [13]. Two remarks have to be made to this subdivision of patients. The first remark: true resistant hypertension can only be confirmed by means of a 24-h ambulatory BP measurement, so there is a possibility that these patients do not have confirmed resistant hypertension. The second remark: as part of the definition of resistant hypertension, patients should use the maximum tolerable dose of their AHDs. This was not explicitly checked as all patients were under control of their physicians with regard to their BPs for at least a year for kidney transplantation patients and approximately half a year for patients at the vascular outpatient clinic.

### Methods of measurement

#### Adherence of antihypertensive drugs

A previously mentioned validated UHPLC-MS/MS method was used to determine 12 AHDs and four of their active metabolites in plasma [10,11]. Only the absence of drug in blood [<lower limit of detection (LLOD)] was used as the criterion for nonadherence [9]. Patient records were used to determine which AHDs were prescribed at the time of the blood collection. When none of the prescribed AHDs was detected in the blood sample, a patient was categorized as completely nonadherent. When at least one of the expected AHDs was detected without the presence of one or more other AHDs, a patient was categorized as partially nonadherent. If all expected drugs were detected, a patient was categorized as adherent.

#### Blood pressure measurements

Blood pressure measurements were performed during every visit to the outpatient clinic as part of standard of care. At the vascular outpatient clinic, BP measurements were performed with an unattended automated office BP measurement (AOBP) (Accutor Plus, Datacope, Paramus, New Jersey, USA). BP was measured for 30–45 min with intervals of 5 min in between the measurements. Measurements were performed with the patient in a seated position and an appropriate sized cuff attached to the nondominant arm.

BP measurements at the nephrology outpatient clinic were performed by means of a single attended office blood measurement according to the Hypertension guidelines [2]. When BPs were higher than expected, an unattended AOBP with three to four measurements was performed to confirm the earlier office BP measurements. When available, AOBP measurements were used for the inclusion of patients and analysis.

### Statistical analysis

We analysed the data in several steps. BP measurements were checked for outliers by means of a boxplot. Values beyond the mean ± 2 standard deviations (SD) were considered outliers, and were double-checked and excluded if they were incompatible with life. For the analysis, only BP values measured during inclusion were used. When blood samples were collected at another moment in time, it was checked if patients still had high BPs, but were not included in the analysis. If the BP was below the inclusion target, patients were excluded from analysis. Normal distribution of the general characteristics from both cohorts was determined with a Shapiro-Wilk test and verified visually for a normal distribution by means of a histogram. Subsequently, characteristics were compared between both cohorts using *t*-test for continuous variables when data were normally distributed and chi-square test for categorical variables. In case of a skewed distribution, a Mann–Whitney test was used for continuous variables.

The difference in adherence between resistant hypertensive patient and non-resistant hypertensive patients was tested by a chi^2^-test. Subsequently, odds ratios were calculated to determine to which extent resistant hypertension was associated with the AHD adherence. A binary multivariate logistic regression analysis was performed to determine the influence of resistant hypertension after adjustment for other covariates, including kidney transplant, age, sex, number of used drugs and serum creatinine.

A posthoc analysis was performed to determine the influence of having a kidney transplantation by subdividing the population by having resistant hypertension and kidney transplant. The same test was performed as described earlier to determine the difference in adherence between the new groups.

We used the SPSS version 24.0 for Windows (IBM Corp, Armonk, New York, USA), Excel version professional plus 2016 software (Microsoft, Redmond, Washington, USA) and GraphPad Prism 9.4 software (GraphPad Software, La Jolla, California, USA) for analysis.

## RESULTS

### General characteristics

A total of 142 patients (64.1% men) was included in the study of which 66 patients were categorized as having ‘resistant hypertension’ (Supplemental material Figure S1). They used an average number of AHDs of 3.2 ± 1.1 of which 85.2% could be measured with our UPLC-MS/MS method (Table 1). Only 12.0% (*n* = 17) of the patients used a combination tablet that combined two or three AHDs (triple pill use: *n* = 11). The median number of used drugs in the resistant hypertension group was 12 (range 4–24) and in the non-resistant hypertension group 9 (range 2–21).

**TABLE 1:** Patient characteristics

	Total (*n* = 142)
Male, *n* (%)	91 (64.1%)
Age (years)	59.8 ± 13.5
BMI (kg/m^2^)	27.8 ± 7.7
CKD-EPI eGFR (ml/min per 1.73 m^2^) [median (IQR)]	50.0 (36.0–70.0)
Creatinine (μmol/l)	135.9 ± 58.8
Resistant hypertension, *n* (%)	66 (46.5)
Mean SBP (mmHg)	155.7 ± 13.6
Mean DBP (mmHg)	84.6 ± 13.1
Diabetes mellitus, *n* (%)	45 (31.7)
Myocardial infarction, *n* (%)	28 (19.7)
Stroke, *n* (%)	13 (9.2)
Atrial fibrillation, *n* (%)	9 (6.3)
Heart failure, *n* (%)	6 (4.2)
Hypercholesterolemia, *n* (%)	15 (10.6)
Mean number of used drugs, *n*	10.7 ± 4.3
Median number of used drugs, (min-max)	10 (2–24)
Mean number of used AHDs, *n*	3.2 ± 1.1
Median number of used AHDs, (min-max)	3 (2–7)
Average measured AHDs of total used AHDs (%)	85.2
Groups used AHDs, *n* (%)	
ACEi	51 (35.7)
ARBs	62 (43.4)
Beta blockers	106 (74.6)
Calcium antagonists	122 (85.9)
Diuretics (one or more)	58 (40.8)
Loop diuretics	16 (11.2)
Thiazide diuretics	36 (25.3)
Aldosterone antagonists	17 (11.9)
Other	6 (4.2)
Doxazosin	32 (22.4)
Registered side effects, *n*	
ACEi	7
ARBs	1
Beta blockers	3
Calcium antagonists	9
Diuretics	4
Doxazosin	3

ACEi, angiotensin-converting enzyme inhibitors; AHD, antihypertensive drug; ARB, angiotensin receptor blocker; CKD-EPI, Chronic Kidney Disease Epidemiology Collaboration; eGFR, estimated glomerular filtration rate; IQR, interquartile range.

Of more than half of the patients (63.4%), samples for adherence determination were collected on the same day they signed informed consent [median = 0 days between signing and sampling, interquartile range (IQR) = 0–49 days]. There was no difference in adherence rates between patients in whom blood was sampled on the day informed consent was signed, and patients in whom blood was sampled at least one day after signing informed consent (*P* = 0.489).

### Adherence of antihypertensive drugs

The overall proportion of patients adherent to AHDs, defined as the presence of all measured AHDs in blood, was 78.2% (*n* = 111 patients). From the nonadherent patients, 87.8% was partially nonadherent and 12.2% nonadherent for all measured AHDs.

A relationship was found between AHD adherence, divided into adherent, partially adherent and nonadherent, and having resistant hypertension [χ^2^(2) = 7.35, *P* = 0.025; Table 2]. Patients with resistant hypertension were less likely to be adherent to ADHs than patients not having resistant hypertension [odds ratio (OR) 0.33; 95% confidence interval (95% CI) 0.14–0.75]. When using multiple logistic regression to correct for other covariates, including number of used drugs, kidney transplantation, age, sex and serum creatinine, patients with resistant hypertension were still more likely to be nonadherent to AHDs, but this was not statistically significant (*P* = 0.078, resistant hypertension (adjusted OR = 0.43; 95% CI 0.17–1.10) (Table 3). This was mainly due to the number of used drugs, which was correlated with resistant hypertension (*r* = 0.27, *P* < 0.001), but no multicollinearity was found by means of the variance inflation factor (VIF = 1.57).

**TABLE 2:** Adherence to antihypertensive drugs in patients visiting the outpatient clinic measured by means of drug concentrations in blood divided by having resistant hypertension

	Adherent, *n* (%)	Partially adherent, *n* (%)	Nonadherent, *n* (%)	Total patients, *n*	*P* (χ^2^(2))
Resistant hypertension (YES)	45 (68.2)	18 (27.3)	3 (4.5)	66	**0.025**
Resistant hypertension (NO)	66 (86.8)	8 (10.5)	2 (2.6)	76	
Total	111 (78.2)	26 (18.4)	5 (3.5)	142	

Bold text indicates values are statistically significant at *P* < 0.05.

**TABLE 3:** Binary logistic regression analysis with (non)adherence as outcome

	Nonadherence vs. Adherence
Resistant hypertension (ref = hypertension)	0.43 (0.17–1.10)
Kidney transplant (ref = No KT)	**3.35 (1.23–9.09)**
Female (ref = Male)	1.21 (0.46–3.23)
Age	1.00 (0.96–1.03)
Serum creatinine	1.00 (0.99–1.00)
Number of used drugs	0.97 (0.86–1.10)
*N*	142
-2 loglikelihood	134.68
Chi-square (df, *P*)	5.96 (8, 0.65)
Nagelkerke R square	0.148

Note: Estimates are odds ratios (ORs) and 95% confidence intervals (95% CI); Bold values are statistically significant at alpha-level of 5%; Cohort dummy consist of two groups: Resistant hypertension and hypertension (reference category). KT, kidney transplant.

**FIGURE 1:**
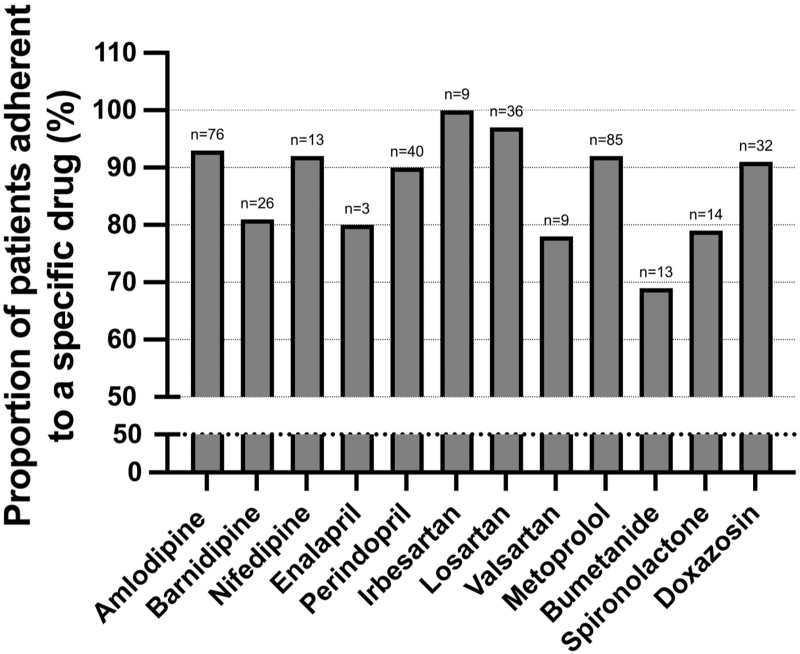
Adherence to antihypertensive drugs in hypertensive patients visiting the outpatient clinic assessed by means of drug concentrations in blood.

When looking at the adherence to individual AHDs, the highest adherence rates were found for irbesartan (100%, *n* = 9), losartan (97%, *n* = 36) and amlodipine (93%, *n* = 76) and the lowest adherence rates for bumetanide (69%, *n* = 13), valsartan (78%, *n* = 9) and spironolactone (79%, *n* = 14) (Fig. 1).

### Posthoc analysis

In the multiple regression analysis for adherence, patients with a kidney transplant were more likely to be adherent to AHDs (Table 3). Therefore, a posthoc analysis was performed to determine the adherence rate when dividing patients based on having a kidney transplant or not. Also, patient characteristics were divided on the basis of having kidney transplant or not (supplemental material Table 1).

When comparing patients with and without kidney transplant, more patients in the non- kidney transplant cohort were nonadherent (partial and total nonadherence combined) compared with the kidney transplant cohort [non-kidney transplant cohort 36.0% vs. kidney transplant-cohort 14.3%, χ^2^(2) = 10.34, *P* = 0.006; supplemental material Table 2]. Furthermore, more patients in the non-kidney transplant cohort fulfilled the definition of resistant hypertension than in the kidney transplant-cohort (non-kidney transplant cohort: resistant hypertension = 62.0% vs. KT cohort: resistant hypertension = 39.1%, Supplemental material Figure S2).

## DISCUSSION

In this study, we investigated the adherence to AHDs in hypertensive patients on treatment with two or more AHDs by measuring drug concentrations in blood. A relatively high adherence rate of 78.2% was found despite our efforts to diminish white-coat adherence by collecting residual blood at a random moment in the year following the signing of an informed consent. This way of collecting blood minimized the awareness of patients on which moment in time their blood sample was used to determine their adherence to AHDs.

The prevalence of adherence to AHDs determined in previous studies that assessed adherence by measuring drug concentrations was lower as compared to our findings. A review by Berra *et al.*[14] showed that adherence rates in patients with uncontrolled BP were between 34 and 77%. Furthermore, eight out of nine presented studies had adherence rates less than 66% [14]. There are several factors that could have contributed to this discrepancy in adherence rates between our study and the studies mentioned in Berra *et al.* [14].

First, it is likely that a large part of the nonadherent patients did not agree to participate in our study, leading to selection bias [15]. Data were collected for research purposes, and therefore, consent of the patients was required. Second, samples were collected during a regular hospital visit, which could have led to a certain degree of white-coat adherence. The time between signing informed consent and drawing of blood did not seem to influence the adherence rate. Third, our UHPLC-MS/MS method was validated to accurately measure 12 AHDs and four active metabolites. These 12 drugs are the most frequently prescribed AHDs in the hospital where the patients were included. However, with the availability of more than 50 different AHDs, it was not possible to set up and validate analytical methods for all prescribed AHDs. This could have led to the exclusion of patients that used AHDs not included in the method or bias in the adherence rate, which could be either positive or negative.

Another factor that could have led to bias, and a higher adherence rate, was the use of office BPs as an inclusion criterion. The use of office BPs is more often associated with ‘white-coat’ hypertension as compared to a 24-h ambulatory BP measurement [16]. Patients with white-coat adherence are more likely to be adherent to their medication, as their elevated BP is due to another mechanism. It was unknown for how many patients participating in this study, this was the case.

When looking at specific AHDs, patients were the least adherent to diuretics (spironolactone and bumetanide) followed by ACE-inhibitors (perindopril and enalapril). This could have been due to side effects, as shown by previous research [17,18]. Also, spironolactone is currently used as fourth line drug [19], probably often prescribed as a consequence of nonadherence to previously prescribed antihypertensives.

Our study included a relatively high number of patients with apparent resistant hypertension and special populations such as patients after kidney transplant. This is due to the fact that patients were recruited at the nephrology and vascular outpatient clinics of a university hospital. Patients with resistant hypertension were more often nonadherent to their antihypertensive medication. This finding is in line with previous studies, and is not surprising, as an increased number of prescribed drugs leads to an increase in nonadherence [1,14]. Furthermore, of all the causes that lead to resistant hypertension, nonadherence is the most difficult to treat [1,20].

In contrast, having kidney transplant was shown to be associated with a higher adherence to AHD therapy. The higher prevalence of adherence measured in patients after kidney transplant is in line with a previously published study of Georges *et al.*[21], which found an overall adherence rate of 79% in a cohort of 53 kidney transplant patients. However, they did not include kidney transplant patients with resistant hypertensions, in whom we also found a higher prevalence of adherence to AHDs as compared to non-kidney transplant patients with resistant hypertension. Remarkably, relatively fewer patients with a kidney transplant fulfilled the definition of resistant hypertension. This is mainly due to the definition of resistant hypertension itself that states that a prescription of at least three AHDs including a diuretic or four AHDs is needed [2]. As diuretics are less often prescribed in kidney transplant recipients, more AHDs (≥4) are needed to fulfil the definition of resistant hypertension. In clinical practice, this is of minor importance because a diagnosis of resistant hypertension does usually not lead to a different treatment. However, when patients are considered for invasive and/or experimental treatment options, for instance, renal denervation or baroreflex activation therapy, they usually have to comply to the criteria for having resistant hypertension [2,22,23]. It should therefore be considered to use a less stringent definition of resistant hypertension for patients with decreased kidney function or after kidney transplant, wherein diuretics are not included. This definition has already been used in clinical studies but has not yet been incorporated in the guidelines [4,13].

In conclusion, the adherence rate to AHDs in hypertensive patients determined with drug concentrations in blood was high with 78.2%. Patients fulfilling the definition of having resistant hypertension had a higher risk of being nonadherent to AHDs. Therefore, healthcare providers should be more aware of the possibility of nonadherence in patients with resistant hypertension and include the assessment of nonadherence to AHDs with an accurate analytical method in the standard management of patients with hypertension. Also, posthoc analysis showed that patients after kidney transplant are more likely to be adherent to AHDs.

## ACKNOWLEDGEMENTS

This study was supported by a ZonMW grant for Rational Pharmacotherapy (Project number 848016003).

J.V. and D.A.H. participated in research design, performance of the research including patient inclusion, writing of the article and critically reviewed and revised the article with special attention for important intellectual content.

L.E.J.P. participated in research design, performance of the research, data collection, writing of the article and revised the article.

C.R.B.R. participated in the performance of the research, collected samples, analysed data and reviewed and revised the article.

S.B. participated in the performance of the research, data analysis and reviewed and revised the article.

B.C.P.K. and T.vG. participated in research design, writing of the article and critically reviewed and revised the article with special attention for important intellectual content.

M.L., D.S., M.W.F.vdH. and M.A.H.S. participated in the performance of the research, including patient inclusion and critically reviewed and revised the article.

All authors approved the final manuscript as submitted and agreed to be accountable for all aspects of the work.

### Conflicts of interest

The authors declare no conflicts of interest.

## Supplementary Material

Supplemental Digital Content
