# Supplementary material for: Monitoring antihypertensive drug concentrations to determine nonadherence in hypertensive patients with or without a kidney transplant
Source: J Hypertens. 2023 May 6;41(8):1239–44. doi: 10.1097/HJH.0000000000003459 (PMC10328507; doi:10.1097/HJH.0000000000003459)

**SUPPLEMENTAL MATERIAL**

**Table 1 Patient characteristics, comparing patients with and without a kidney transplantation (KT)**

|  | **Non-KT cohort (n=50)** | **KT cohort (n=92)** | **Significance: p-value <0.05** |
| --- | --- | --- | --- |
| **Male, n (%)** | 27 (54.0%) | 64 (68.8%) | 0.065 |
| **Age, year** | 61.3 ± 13.5 | 59.0 ± 13.5 | 0.337 |
| **BMI, kg/m^2^** | 25.3 ± 10.6 | 29.3 ± 5.0 | **0.018** |
| **CKD-EPI eGFR, mL/min/1.73m^2^ (median (IQR))** | 55.5 (31.0-76.8) | 46.5 (37.0-61.0) | 0.283 |
| **Creatinine, µMol/L** | 130.4 ± 67.9 | 139.1 ± 53.3 | 0.387 |
| **Resistant hypertension, n (%)** | 31 (62.0) | 35 (38.0) | **0.006** |
| **Mean SBP, mmHg** | 158.7 ± 15.7 | 154.0 ± 12.1 | 0.071 |
| **Mean DBP, mmHg** | 87.1 ± 14.3 | 83.2 ± 12.2 | 0.092 |
| **Mean years after kidney transplantation, year**  **Missing data (n)** | NA | 7.6 ± 4.8  1 |  |
| **Living kidney donor, n (%) Missing data** | NA | 73 (80.2%)  1 |  |
| **Diabetes mellitus, n (%)** | 13 (26.0) | 32 (34.8) | 0.283 |
| **Myocardial infarction, n (%)** | 9 (18.0) | 19 (20.7) | 0.704 |
| **Stroke, n (%)** | 8 (16.0) | 5 (5.4) | **0.037** |
| **Atrial Fibrillation, n (%)** | 3 (4.0) | 7 (7.6) | 0.399 |
| **Heart Failure, n (%)** | 2 (4.0) | 4 (4.3) | 0.922 |
| **Hypercholesterolemia, n (%)** | 10 (20.0) | 5 (5.4) | **0.006** |
| **Mean number of used drugs, n**  **Median number of used drugs (min-max)** | 8.8 ± 3.9  8 (2-18) | 11.7 ± 4.2  12 (4-24) | **<0.001** |
| **Mean number of used AHDs, n**  **Median number of used AHDs (min-max)** | 3.6 ± 1.3  4 (2-7) | 3.0 ± 1.0  3 (2-6) | **0.003** |
| **Average measured AHDs of total used AHDs (%)** | 80.2 | 88.0 | **0.012** |
| **Groups used AHDs, n (%)** ACEi  ARBs  Beta blockers  Calcium antagonists  Diuretics  Other (including doxazosin) | 20 (40.0) 25 (50.0) 35 (70.0) 44 (88.0) 30 (60.0) 16 (32.0) | 31 (33.3) 37 (39.8) 71 (77.2) 78 (84.8) 28 (30.4) 24 (25.8) | 0.455 0.262 0.348 0.599 **<0.001**  0.454 |
| **Registered side effects, n** ACEi  ARBs  Beta blockers  Calcium antagonists   Diuretics  Doxazosin | 4  1  3  6  2  0 | 3  0  0  3  2  3 |  |

*ACEi = angiotensin converting enzyme inhibitors, AHD = antihypertensive drug, ARB = angiotensin receptor blocker, BMI = body mass index, CKD-EPI = Chronic Kidney Disease Epidemiology Collaboration, DBP = diastolic blood pressure, eGFR = estimated glomerular filtration rate, IQR = interquartile range, KT = kidney transplantation, SBP = systolic blood pressure*

**Table 2** Adherence to antihypertensive drugs in patients visiting the outpatient clinic measured by means of drug concentrations in blood divided by having kidney transplantation.

|  | **Adherent, n (%)** | **Partially adherent, n (%)** | **Non-adherent, n (%)** | **Total patients, n** | **p-value (χ^2^(2))** |
| --- | --- | --- | --- | --- | --- |
| **Kidney transplantation (YES)** | 79 (55.6) | 12 (8.5) | 1 (0.7) | 92 | **0.006** |
| **Kidney transplantation (NO)** | 32 (22.5) | 14 (9.9) | 4 (2.8) | 50 |
| Total |  | | | 142 |  |

**Figure legends**

**S1 Inclusion flowchart of patients in RHYME-AD**

**
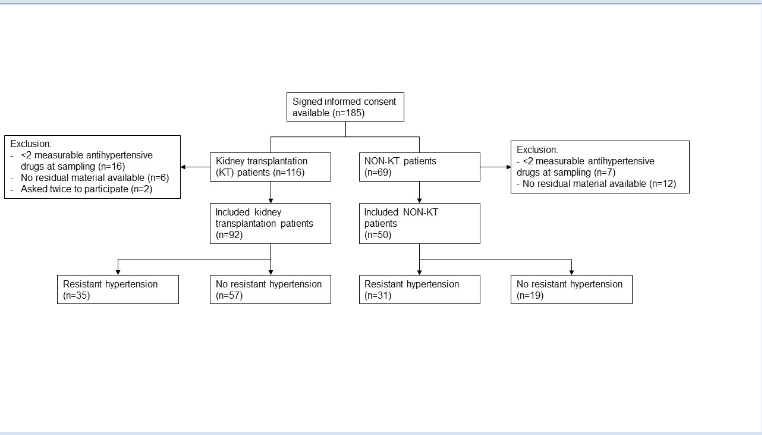
**

**S2 Prevalence of adherence to antihypertensive drugs measured by drug concentrations in blood in patients with and without resistant hypertension (YES/NO) and/or with and without kidney transplantation (YES/NO)**

**combination of partial and total non-adherence to antihypertensive drugs*


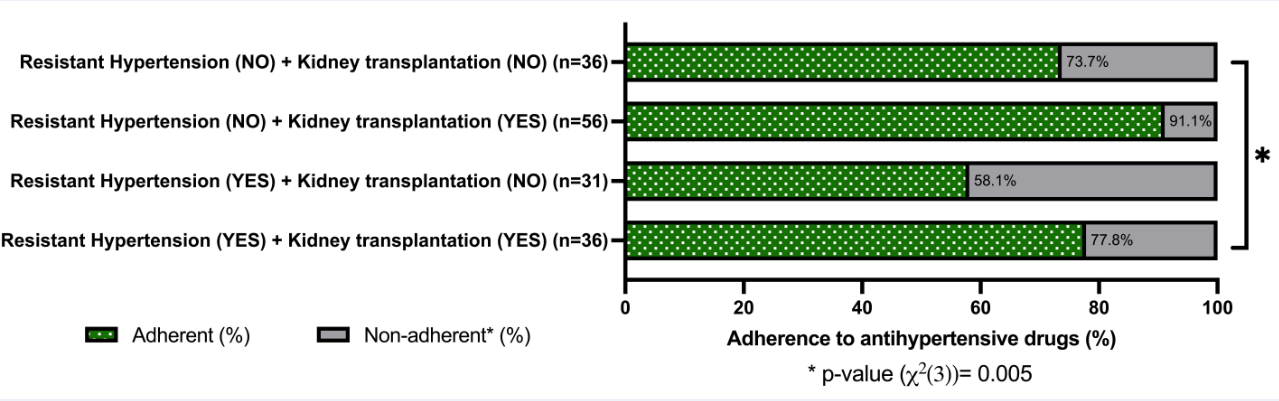

Supplement: Supplemental Digital Content [file jhype-41-1239-s001.doc]
